# Type 1 Insulin-Like Growth Factor Receptor Nuclear Localization in High-Grade Glioma Cells Enhances Motility, Metabolism, and *In Vivo* Tumorigenesis

**DOI:** 10.3389/fendo.2022.849279

**Published:** 2022-04-27

**Authors:** Ayelen Martin, María Celia Fernandez, Elizabeth R. Cattaneo, Claudio D. Schuster, Marcela Venara, Florencia Clément, Ariel Berenstein, Mercedes García Lombardi, Ignacio Bergadá, Mariana Gutierrez, Marcelo A. Martí, María R. Gonzalez-Baro, Patricia A. Pennisi

**Affiliations:** ^1^ Centro de Investigaciones Endocrinológicas “Dr. César Bergadá” CONICET—FEI—División de Endocrinología, Hospital de Niños R. Gutierrez, Buenos Aires, Argentina; ^2^ Instituto de Investigaciones Bioquímicas de La Plata, CONICET, Facultad de Ciencias Médicas, Universidad Nacional de La Plata, La Plata, Argentina; ^3^ Departamento de Química Biológica, Facultad de Ciencias Exactas y Naturales, Universidad de Buenos Aires (FCEyN-UBA) e Instituto de Química Biológica de la Facultad de Ciencias Exactas y Naturales (IQUIBICEN) CONICET, Pabellòn 2 de Ciudad Universitaria, Ciudad de Buenos Aires, Argentina; ^4^ Instituto Multidisciplinario de Investigaciones en Patologías Pediátricas, CONICET, Hospital de Niños R. Gutierrez, Buenos Aires, Argentina; ^5^ Servicio de Oncología, Hospital de Niños R. Gutierrez, Buenos Aires, Argentina

**Keywords:** OSI906, linsitinib, cell metabolism, nuclear IGF1R, high-grade glioma

## Abstract

Gliomas are the most frequent solid tumors in children. Among these, high-grade gliomas are less common in children than in adults, though they are similar in their aggressive clinical behavior. In adults, glioblastoma is the most lethal tumor of the central nervous system. Insulin-like growth factor 1 receptor (IGF1R) plays an important role in cancer biology, and its nuclear localization has been described as an adverse prognostic factor in different tumors. Previously, we have demonstrated that, in pediatric gliomas, IGF1R nuclear localization is significantly associated with high-grade tumors, worst clinical outcome, and increased risk of death. Herein we explore the role of IGF1R intracellular localization by comparing two glioblastoma cell lines that differ only in their IGF1R capacity to translocate to the nucleus. *In vitro*, IGF1R nuclear localization enhances glioblastoma cell motility and metabolism without affecting their proliferation. *In vivo*, IGF1R has the capacity to translocate to the nucleus and allows not only a higher proliferation rate and the earlier development of tumors but also renders the cells sensitive to OSI906 therapy. With this work, we provide evidence supporting the implications of the presence of IGF1R in the nucleus of glioma cells and a potential therapeutic opportunity for patients harboring gliomas with IGF1R nuclear localization.

## Introduction

Central nervous system (CNS) tumors are the most frequent solid tumors in the pediatric population (1). Gliomas are the most represented group (40–50%), followed by medulloblastomas and ependymomas (2). The survival for these pathologies has increased in the past few years, but patients still suffer sequels due to treatments (3). Adult-type diffuse gliomas are likewise the most common malignant primary brain tumors in adults and are refractory to conventional therapy, including surgical resection, radiotherapy, and chemotherapy (4). Thus, the search for new prognosis factors that would allow treatment individualization is a priority.

The WHO grading of gliomas was based on their histological characteristics and natural history and divided them into four grades of malignancy (1–4). In 2016, this classification was updated in order to include molecular findings such as diagnostic biomarkers and prognostic factors (5). While preparing this manuscript, the fifth edition of the WHO Classification of Tumors of the CNS (CNS5) was delivered, introducing major changes in the use of molecular diagnostic in tumor classification (6). Though CNS5 features substantial changes to advance the role of molecular diagnostics in CNS tumor classification, it still remains rooted in other established approaches to tumor characterization, including histology and immunohistochemistry. It is to be noted that the term “glioblastoma” is no longer used in the setting of a pediatric-type neoplasm.

Particularly, most pediatric gliomas are low grade (1 and 2) and do not develop into high-grade (3 and 4) tumors as those in adults do (7). For pediatric gliomas, molecular biomarkers with prognostic value are scarce (8, 9). Hence, the search for new biomarkers of prognosis value or that could lead to treatment individualization is a priority.

It has already been established that gliomas follow multiple metabolic strategies to sustain cellular growth (10). On the one hand, glucose metabolized by aerobic glycolysis (incomplete and non-oxidative metabolism of glucose in the presence of oxygen, also known as “Warburg effect”) contributes not only to energy production but also by leaving intermediates of the tricarboxylic acid cycle (TCA) available for different anabolic pathways (10). On the other hand, fatty acids, which can be obtained from the bloodstream or built from carbon chains within the glioma cell, also act as critical bio-energetic substrates (11).

The insulin and insulin-like growth factor (IGF) system of ligands (IGF1 and IGF2) and receptors (IGF1R and IR) plays important physiological roles to ensure the cellular survival and proliferation of many tissues (12). Additionally, several components of the IGF system are known to be present in tumors from both adults and pediatric patients (13). Particularly, IGF1R is a membrane receptor belonging to the tyrosine kinase family of receptors which fulfill its functions mainly by phosphorylation of intracellular molecules, including the ras-raf-MAPK and PI3K-AKT signaling cascades (14). Conventional inositide-specific phospholipase C (PI-PLC) β1 activation and Small G protein, Rac1, Rho1, and CDC42 protein activation had also been reported to be promoted by IGF stimulation (15). More recently, it has become clear that receptor endocytosis is also crucial for the formation of signaling complexes, for the fine-tuning of hormonal signals, and for proper receptor homeostasis and regulation (16). It has been previously shown that the presence of functional IGF1R is needed for the acquisition of a neoplastic phenotype in different cells (17). Moreover, its overexpression has been demonstrated in different cancers such as breast (18) and pediatric adrenocortical tumors (19). In 1997, Glick et al. provided the first evidence of the presence of IGF receptors in human CNS tumors (20).

Despite being a transmembrane receptor, intact IGF1R has been detected in the nuclei of human malignant and non-malignant cells (21, 22). IGF1R phosphorylation and the subsequent SUMOylation of three conserved lysins (Lys1025, Lys1100, and Lys1120) of the mature protein are requisite for nuclear receptor translocation (22). Although IGF1R function in the nucleus is still unknown, it has already been associated with poor prognosis in patients with breast cancer (23), anaplastic lymphoma (24), and synovial sarcomas (25). There is also a study showing that IGF1R nuclear staining is a predictive biomarker for therapy response in sarcomas (26). In a previous study conducted by our group in which tumor samples from pediatric gliomas were analyzed, we have demonstrated that IGF1R nuclear localization is associated with higher-grade tumors and also related with worst clinical outcome of those patients (27).

The present study was designed to explore the role of IGF1R intracellular localization in the biology of pediatric high-grade glioma *in vitro* and, using subcutaneous xenografts as a proof of concept, by *in vivo* studies. We used the U87 cell line to develop a model in which IGF1R was able (U87WT) or unable (U87Mut cells) to translocate to the nucleus. Our results suggest that IGF1R nuclear localization may contribute to an aggressive phenotype in glioblastoma cells by increasing their motility and metabolism rather than stimulating their proliferation.

## Materials and Methods

### Site-Directed Mutagenesis

The pEGFP vector containing mature IGF1R protein coding sequence as a fusion protein with GFP (GFP-IGF1R) was kindly provided by Dr. Rosemary O’Connor (Department of Biochemistry, University of Cork, Ireland). Lysine residues—Lys1025, Lys1100, and Lys1120—were changed to arginines to prevent IGF1R SUMOylation and translocation to the nucleus by site-directed mutagenesis to obtain the GFP-IGF1R^1025x-1100x-1120x^ vector for the experiments. Q5 high-fidelity DNA polymerase (New England Biolabs, Ipswich, MA, USA) and specific primers were used to perform mutations, which were verified by DNA sequencing in ABI PRISM 310 Genetic Analyzer (Applied Biosystems, Foster City, CA, USA).

### Cell Line

The U-87MG (U87) human glioblastoma cell line was purchased from American Type Culture Collection (Manassas, VA, USA). Cells were cultured in Minimum Essential Medium Eagle (MEM) supplemented with 10% fetal bovine serum (FBS) and maintained at 37°C in a humidified 5% CO_2_ environment.

### Stable Transfection and Selection of Cell Lines

The U87 human glioblastoma cells were transfected with an empty vector containing green fluorescent protein (U87Empty). To generate IGF1R-overexpressing clones, the cells were transfected with GFP-IGF1R and GFP-IGF1R^1025x-1100x-1120x^ vectors. All experiments were performed using Lipofectamine 3000 (Invitrogen, Carlsbad, CA, USA) following the manufacturer’s protocols. Stable clones were selected using G418 antibiotic (Sigma Aldrich, St Luis, MO, USA), and once established, RNA and proteins were obtained as previously described (27) to test the IGF1R expression levels.

### IGF1R Inhibition OSI906

To test the specificity to IGF1 stimulation, cells were pre-incubated with OSI906, a dual IGF1R/IR inhibitor (Selleckchem, Houston, TX, USA), for 60 min and throughout the time of IGF1 stimulation. The concentration range was 0.25 to 1 µM, as has been described previously (28).

### IGFI Stimulation

Cells were seeded on six-well plates, allowed to grow up to 70% confluence, and starved overnight with serum-free medium. On the next morning, the cells were stimulated for 10 min with 50 nM IGF1, with or without preincubation with crescent concentrations of IGF1R inhibitor OSI906. The cells were harvested on lysis buffer containing protease inhibitors, and proteins were extracted as previously described (29) to study IGF1R, AKT, and MAPK activation.

### IGF1R Nuclear Translocation

To study IGF1R subcellular localization, cells were seeded on six-well plates (fractionation) or over 12-mm coverslips (immunofluorescence), allowed to grow up to 70% confluence, and starved overnight with serum-free medium. On the next day, the cells were stimulated for 8 h with 50 nM IGF1, with or without preincubation with 0.5 µM OSI906. The cells incubated with serum-free media were used as the negative control. After incubation, the cells were washed with phosphate-buffered saline (PBS) and processed for posterior studies as follows:

–Immunofluorescence studies (IF): Cells were fixed with 4% paraformaldehyde, incubated with Hoechst (5 µg/ml) for 30 m, washed with PBS, and mounted using Vectashield mounting media (Vector Laboratories, CA, USA). Images were obtained using Olympus FV1000 confocal microscope and analyzed with Fluoview FV1000 software.
–Subcellular fractionation: To prepare cytosolic and nuclear extracts, cells grown on six-well plates were trypsinized and centrifuged, and the pellet was resuspended in a buffer solution (containing 10 mM EDTA, 1% IGEPAL, 1 mM DTT, and protease inhibitors) and incubated for 10 min on ice. Next, the cells were centrifuged at high speed for 10 min at 4°C, and the supernatant containing the non-nuclear fraction was saved at -70°C. The pellets were washed and resuspended in a second buffer (20 mM Tris, 1% SDS, 5 mM EGTA, 0.5% Triton, 150 mM NaCl, and protease inhibitors), sonicated, and centrifuged at high speed for 10 min at 4°C to obtain the supernatant containing the nuclear extracts.

### Proliferation Assay

Cells were seeded on 24-well plates in complete medium (MEM 10% FBS) and allowed to attach overnight. One plate was used for each point (day) of the assay and each condition (with or without IGF1) in triplicate, plus an additional plate that was used to determine the initial number of cells (time zero plate). At 24 h after seeding, the cells attached to the time zero plate were harvested by trypsinization and counted after 0.5% trypan blue staining. In the remaining plates, the medium was replaced by MEM supplemented with 1% FBS, with or without IGF1, to a final concentration of 50 nM. The cells were cultured over 1, 2, or 4 days, trypsinized, and counted.

### Cell Cycle by Flow Cytometry

Cells were starved in serum-free medium overnight and stimulated for 24 h with 1% FBS MEM, with or without 50 nM IGF1, or 10% FBS as control of proliferating cells. After culture, the cells were fixed in 66% cold ethanol in PBS at 4°C overnight and then stained for 30 min in the dark with a solution containing 50 µg/ml RNase A and 20 µg/ml of PI. Finally, the stained cells were analyzed by using FACSCalibur flow cytometer (Becton Dickinson, San Jose, CA, USA), and data were processed by Flowing Software (Perttu Terho, Turku Centre for Biotechnology).

### Cell Migration Assay

Cells were seeded on six-well plates in complete medium and were grown to completely cover each well surface. The monolayer was scraped in a straight line to create a scratch with a p200 pipet tip, cell debris was removed by washing with PBS, and media was replaced by MEM supplemented with 1% FBS, with or without 50 nM IGF1. Time zero images from a specific point of the scratch were taken, and the same field images were taken 36 h later. To verify specificity in response to IGF1, the cells were preincubated with OSI906. The experiments were performed in triplicates.

### Measurement of Lactate Dehydrogenase Activity

Cells were seeded on 24-well plates and cultured for 24 h, with or without 50 nM IGF1, and preincubated with OSI906 to verify the IGF1-specific response. After this period, the cells were washed and sonicated in saline solution, centrifugated to obtain a clean solution, and measured in COBAS C311 autoanalyzer (Roche Diagnostics, IN, USA). The lactate dehydrogenase (LDH) activity values obtained were relativized to milligrams of DNA per well. The experiments were performed in triplicates.

### Lipid Droplets

Lipid droplets were visualized by oil red O (ORO) staining as previously described (30). Briefly, cells were cultured for 24 h in 8-well chamber slides, with or without 50 nM IGF1. After incubation, they were washed and fixed with 10% v/v formalin for 1 h and stained with ORO solution. Background was removed, and the cells were counterstained with hematoxylin. The lipid droplets were analyzed using Eclipse 50i microscope (Nikon Instruments, Melville, NY, USA).

### Thin-Layer Chromatography: Metabolic Labeling and Phospholipid Analysis

To study *de novo* phospholipid synthesis, cells were seeded in 6-well plates, treated with IGF1 with or without preincubation with OSI906 as described above and labeled with 0.25 μCi/ml of 1^-[14C]^ acetic acid in MEM without FBS. After 24 h, the medium was removed, and monolayers were washed with ice-cold PBS and scraped in 2 consequent additions of 0.5 ml cold methanol. Then, 0.5 ml chloroform and 0.25 ml H_2_O were added to complete the solvent system, and the lipids were extracted as described by Bligh and Dyer (31). Finally, total lipids were spotted on precoated thin-layer chromatography glass plates (Silica Gel 60, Merck, Darmstadt, Germany) and resolved with hexane/ethyl ether/acetic acid (80:20:2 by volume) as a solvent system for neutral lipid separation. Pure lipid standards were used to identify the lipids. Standards were visualized under iodine vapor, and the radioactivity associated with each spot was quantified in Storm 840 scanner (GE Healthcare, United Kingdom).

### Western Blotting

Once whole cell or nuclear and non-nuclear protein fractions were obtained, the protein concentration in the supernatants was determined using Bradford reagent. Fifty micrograms of proteins was resolved by SDS-PAGE and transferred to polyvinylidene fluoride membranes. Blots were blocked and probed with various antibodies: anti-IGF1R β (#3027), anti-pIGF1R β (#3918), anti-AKT (#9272), anti-pAKT (#9271), anti-MAPK 42-44 (#9102), anti-pMAPK 42-44 (#9101), anti-S6K (#2708), anti-pS6K (# 9205), and anti-β actin (#4970) from Cell Signaling Technology (Boston, MA, USA); anti-lamin B (SC-6217) from Santa Cruz Inc. (Dallas, TX, USA); anti-pPDC subunit E1-α (NB110-93479) and ACSL5 (H00051703-M01) from Novus Biologicals (Centennial, CO, USA); anti-FAS (610962) from BD Biosciences (San Jose, CA, USA); and anti-GAPDH (MAB374) from Millipore (Darmstadt, Germany).

### Real-Time PCR

Cell RNA was obtained using Direct-Zol RNA Kit (Zymo Research, Irvine, CA, USA), following the manufacturer’s protocol. RT-PCR was performed using 500 ng of RNA of each sample with random hexamers to prime the reverse transcription catalyzed by Super Script II (Invitrogen, Carlsbad, CA). The resulting cDNA was diluted by 1:10, and 3 μl from each dilution was subjected to rqPCR in triplicates using Kapa Syber Fast qPCR master mix (Kapa Biosystems, Boston, MA) in Step One Plus Real-Time PCR System (Life Technologies, Carlsbad, CA, USA). Several targets such as IGF1, IGF2, IGF1R, IR, GLUT1, CCTα, and diacylglycerol O-acyltransferase 1 (DGAT1) were studied and normalized to TATA box binding protein (TBP) as internal control (primer sequences are available upon request); the mRNA values were calculated using relative quantitation method and are presented as fold change compared to control conditions.

### Microarray Data Analysis

The derived RNA from U87, U87WT 5, and U87Mut 5 cells was hybridized to a high-density oligonucleotide SurePrint G3 Human Gene Exp v3 array plate (Agilent Technologies, Santa Clara, CA). Microarray expression data were first background-corrected and normalized within and between arrays using the limma package. The corresponding expression data in arbitrary units for each gene, in each condition, is available as supplementary information. The normalized expression data was further simplified to a trinary matrix with values of 1 (for overexpressed genes), 0 (no change), and -1 (underexpressed genes), using the previously obtained fold change values. Genes were considered over/underexpressed when their expression data value was above/below 0.5 SD. The SD was computed using as sample all genes in each sample separately. The results were further analyzed by building gene lists that are differentially expressed between experimental conditions (FBS free medium, IGF-1 50 nM, OSI+IGF-1), performed with an in-house python script. Specifically, we made the following comparisons: genes overexpressed or underexpressed after IGF1 stimulation (IGF1 50nM) and genes over or underexpressed in all conditions.

The resulting gene lists were used as input for the DAVID database in order to analyze pathway enrichment.

### Glioblastoma Xenografts

Immunodeficient mice [N:NIH (S)- Fox 1^nu^] were housed in standard conditions of 12-h light/12-h dark cycle with water and food *ad libitum*, in accordance with the National Institutes of Health guide for the care and use of laboratory animals (32).

Six-week-old male mice were injected subcutaneously on the right flank with 1.5 × 10^6^ U87 empty cells or U87WT 5 and U87Mut 5 clones. The incidence and latency period of tumor development were recorded up to 25 days after inoculation.

The two longest perpendicular axes in the x/y plane of each developed tumor were measured daily using a vernier caliper for tumor volume calculation (33). At 3 h before sacrifice, the mice received an intraperitoneal BrdU injection (30 mg/kg body weight) to assess cell proliferation by *in vivo* BrdU incorporation. The tumors were dissected and prepared for histological analysis.

### OSI906 treatment

The mice were given 75 mg/kg of OSI906 orally by gavage on days 0, 3, and 6 once tumors developed by U87WT 5 or U87Mut 5 cells reached a volume of 150 mm^3^. The tumors were measured daily using a caliper as previously described (33).

### Sample Size Calculation

Sample size calculation was performed to establish the least number of mice to be injected to detect differences in tumor volumes. We calculated that we would need to assign 5 animals in each group to have 90% power to detect a mean difference of 50 mm^3^ of tumor volume (mean) between groups, at the two-sided *P*-value of 0.05. The minimally important difference was estimated on the basis of a pilot study, in which an average of 50 mm^3^ of volume difference could be detected between tumors from WT (WT5) and mutant (Mut5) cells at 14 days after injection.

All animals were treated and cared for in accordance with standard international animal care protocols. All procedures were approved by the Animal Care and Use Committee of the Hospital de Niños Dr. R Gutierrez (DI-2012-253-HGNRG).

### Statistical Analysis

Mann–Whitney *U* nonparametric test was used to compare data between groups (2 conditions). ANOVA, followed by *post-hoc* tests, was used for experiments with multiple comparisons. Kaplan– Meier curves were used to analyze the differences in incidence and latency period between U87-, U87WT-, and U87Mut-injected cells. The results are reported as mean ± standard deviation, unless indicated otherwise. Statistical significance was defined as a *p*-value of less than 0.05 (*P* < 0.05). All the statistical analysis and the graphs presented were done using GraphPad Prism software (Windows version 7.01, GraphPad Software, La Jolla, CA, USA; http://www.graphpad.com).

## Results

### Characterization of U87 Cell Lines Overexpressing Wild-Type and Mutant IGF1R

**Figure 1:**
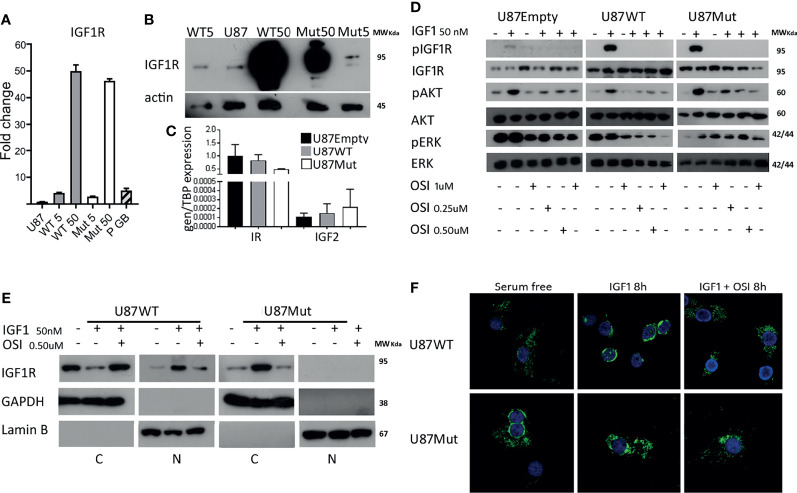
Characterization of wild type and mutant IGF1R U87 clones. **(A)** IGF1R mRNA expression in clones generated after the transfection of wild-type (WT) or mutant (Mut) IGF1R in U87 cells. Values are presented as fold change compared to U87 cells. **(B)** Representative western blotting for IGF1R in protein extracts from WT and Mut clones. **(C)** The IR and IGF2 mRNA expression in U87Empty, U87WT 5, and U87Mut 5 cells was calculated using relative quantitation method. Values are presented relative to TBP mRNA expression. **(D)** Western blotting of U87Empty, U87WT 5, and U87Mut 5 cells after 10 min of 50-nM IGF1 stimulation and/or pre-incubation (1 h) with IGF1R/IR inhibitor OSI906 (OSI) in different concentrations (0.25, 0.5, and 1 μM). The membranes were blotted with antiphospho-IGF1R (pIGF1R), antiphospho-AKT (pAKT), or antiphospho-ERK (pERK). The blots were stripped and reprobed with antitotal-IGF1R (IGF1R), antitotal-AKT (AKT), or antitotal-ERK (ERK). **(E)** Subcellular fractionation of U87WT 5 and U87Mut 5 cells after 8 h of 50-nM IGF1 stimulation with or without pre-incubation (1 h) with 0.5 μM OSI906 (OSI) followed by Western blotting. GAPDH and Lamin B were used as markers of non-nuclear and nuclear fractions, respectively. **(F)** Confocal immunofluorescence microscopy of U87WT 5 and U87Mut 5 cells after 8 h of 50-nM IGF1 stimulation with or without pre-incubation (1 h) with 0.5 μM OSI906 (OSI). Representative pictures; IGF1R in green (pEGFP vector) and nucleus in blue (Hoechst staining).

To characterize the impact of IGF1R nuclear localization, we used a vector containing a mature IGF1R protein coding sequence as a fusion protein with GFP (IGF1R-GFP) and generated a mutant receptor that is not able to translocate to the cell nucleus (GFP-IGF1R^1025x-1100x-1120x^) by site-directed mutagenesis. After the selection of U87 cells using geneticin (G418), we obtained stably transfected clones expressing 5 or 50 times wild-type mRNA-IGF1R (WT 5 and WT 50 clones) and 5 or 50 times mRNA for IGF1R mutant protein (Mut 5 and Mut 50 clones, **Figure 1A**). WT 5 (U87WT 5) and Mut 5 (U87Mut 5) cells had comparable levels of IGF1R mRNA expression than those observed in samples from high-grade gliomas from pediatric patients (**Figure 1A**, P GB) that we used as a reference for expression levels, while U87WT50 and U87Mut 50 expressed much higher mRNA levels of IGF1R. The overexpression of IGF1R was confirmed in all clones by protein Western blot analysis (**Figure 1B**). We also studied the expression of other components of the IGF system: Insulin receptor (IR) expression was higher than IGF1R expression in U87Empty cells and did not change in wild-type or mutant IGF1R-expressing clones. As for ligands, IGF1 expression was not detectable, while a very low expression of IGF2 was found in all cell lines (**Figure 1C**).

Considering IGF1R expression in U87 cell line and its clones shown in **Figure 1A**, we decided to continue our work with U87WT5 and U87Mut5 cells rather than with the other 2 cell clones. Though we are aware of the limitations of the model, we considered that the chosen clones better represented the pediatric gliomas regarding mRNA IGF1R expression levels. WT 5 (U87WT 5) and Mut 5 (U87Mut 5) clones will be referred, from now on, as U87WT and U87Mut. Next, we characterized the response to a short IGF1 stimulation. Phosphorylated IGF1R and activated downstream pathways are shown in **Figure 1D**. To verify the specificity of the response to IGF1 stimulus, we preincubated the cells for 1 h with OSI906, a dual inhibitor for IGF1R and IR (28). We performed a dose–response curve and selected the dose of 0.5 µM OSI906, where all clones showed inhibition of IGF1R phosphorylation and decreased pAKT and pMAPK levels (**Figure 1D**).

By confocal IF studies, we analyzed IGF1R cellular localization after 8 h of IGF1 stimulation in U87WT and U87Mut clones. We found that, in U87WT, IGF1R was able to translocate to the nucleus, while this effect was not observed for mutants (**Figure 1F**). The specificity of the response was verified by pre-incubating the cells for 1 h with OSI906, which inhibits receptor translocation in U87WT. The observed results were confirmed by subcellular fractionation, followed by Western blotting (**Figure 1E**).

### IGF1R Nuclear Localization Increases Motility But Has No Effects Over Glioma Cell Proliferation

**Figure 2:**
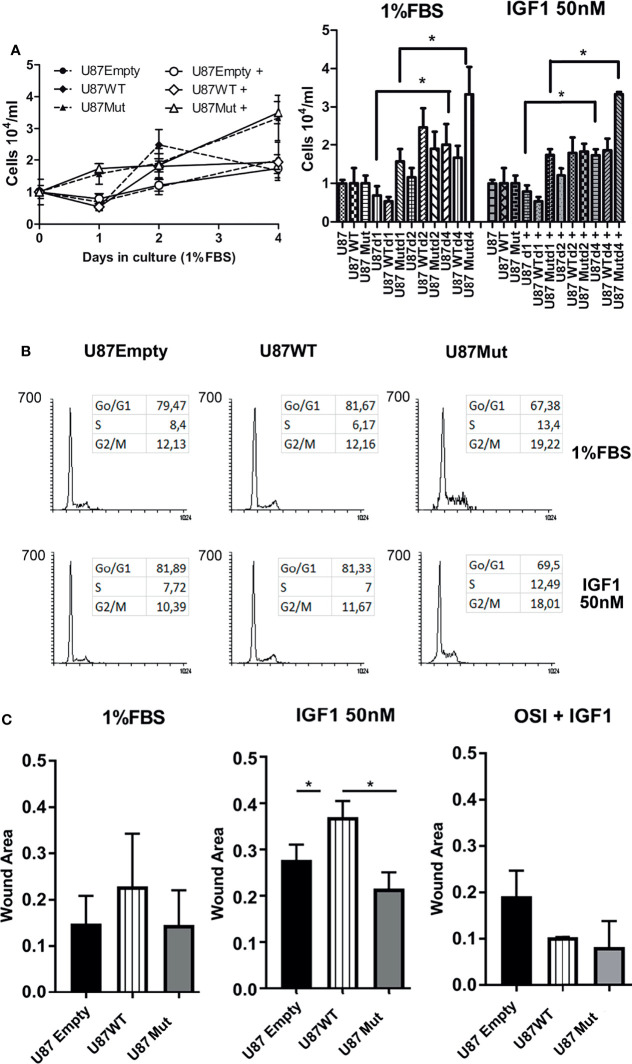
Effects of IGF1R localization over the proliferation and migration of glioma cells. **(A)** Left: U87Empty (solid circles), U87WT (solid rhomboids), and U87Mut (solid triangles) cells were grown for 4 days with low serum concentration (1% FBS) or in the presence of 50 nM IGF1 [U87Empty + (open circles), U87WT+ (open rhomboids), and U87Mut+ (open triangles)]. The results of a representative experiment from a total of three performed in triplicates are shown; Right: total number of cells, according to cell line, days in culture, and treatment. Values are expressed as mean ± SD (*p < 0.05, ANOVA, Tukey’s post-test). **(B)** U87Empty, U87WT, and U87Mut cells were cultured 24 h in low-serum conditions (1% FBS) with or without 50 nM IGF1 stimulus, fixed, stained with PI, and analyzed by flow cytometry. A representative experiment is shown. **(C)** Wounding assays were performed to U87Empty, U87WT, and U87Mut cell confluent cultures. The cells were grown for 36 h in low-serum conditions (1% FBS) or in the presence of 50 nM IGF1. To test the specificity of the response, the cells were pre-incubated (1 h) with 0.5 μM OSI906 (OSI+IGF1). The results obtained from three independent experiments are presented as the proportion of covered area related to time 0. Values are expressed as mean ± SD (*p < 0.05, ANOVA, Tukey’s post-test).

When cultured in complete medium (10% FBS), U87Empty cells and both U87WT and U87Mut clones showed a sustained exponential growth (data not shown). We performed proliferation assays for 4 days in cells cultured with a low serum concentration (1% FBS) or in the presence of 50 nM IGF1 (**Figure 2A**, left). After 4 days in culture, both U87 and U87Mut cells reached a significantly higher number of cells, compared to day 1 (U87d 1 *vs*. U87d4; U87d1+ *vs*. U87d4+; U87Mutd1 *vs*. U87Mutd4, U87Mutd1+ *vs*. U87Mutd4+; *p* < 0.05, ANOVA, Tukey’s post-test), while no differences were observed in U87WT cells. Interestingly, there were no differences between cells cultured under 1% FBS *vs*. 50 nM IGF1 at any day tested (*p* = ns, Mann–Whitney test, **Figure 2A**, right). In accordance with this result, no changes in cell cycle progression were found after 24 h of incubation with IGF1 (**Figure 2B**).

As IGF1 has known effects modulating cell motility and invasion (29, 34), we decided to check if cell migration was affected by the ability of IGF1R to translocate to the nucleus. Cell motility, assessed by wounding assay, was increased after 36 h of IGF1 stimulation in U87WT clones compared to U87Empty or U87Mut cells. This effect was abrogated by preincubation for 1 h with OSI906 (**Figure 2C** and **Supplementary Figure 1**).

### IGF1R Nuclear Localization Stimulates Glioma Cell Metabolism by Increasing Glucose Uptake and the Novo Lipid Synthesis

**Figure 3:**
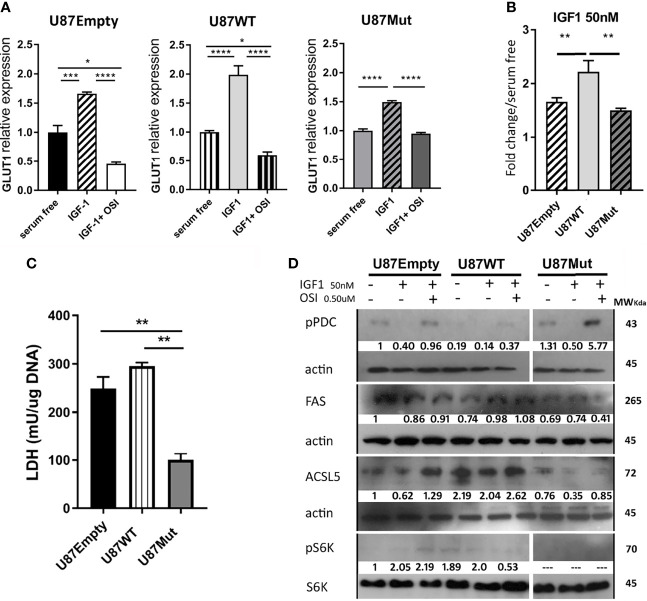
Effects of IGF1R localization over cell metabolism. **(A)** U87Empty, U87WT, and U87Mut cells were cultured 24 h in serum-free medium or with 50-nM IGF1 stimulation (IGF1). To test the specificity of the response, pre-incubation (1 h) with 0.5 μM OSI906 was also performed (IGF1+ OSI). **(A, B)** GLUT1 mRNA expression was calculated by rqPCR by the relative quantitation method. **(A)** Values are presented as fold change compared to control conditions (serum-free). **(B)** IGF1 stimuli comparison between cell lines. The results are presented as fold change due to IGF1 stimulation over basal conditions (serum-free). Values are expressed as mean ± SD of three independent experiments performed in triplicates (*p < 0.05, **p < 0.005, ***p < 0.001, ****p < 0.0001, ANOVA, Tukey’s post-test). **(C)** LDH enzyme activity was measured and normalized to DNA content (mg). The results are expressed as mean ± SD of three independent experiments (**p < 0.005, ANOVA, Tukey’s post-test). **(D)** Representative western blotting (*n* = 3) for U87Empty, U87WT, and U87Mut cell protein extracts. The membranes were blotted with anti-pPDC (line 1), anti-FAS (line 3), anti-ACSL5 (line 5), and anti-pS6K (line 7). Each blot was stripped and reprobed with anti-β actin (lines 2, 4, and 6) or antitotal-S6K (line 8). The relative quantification of the bands is shown under each line.

The IGF system has an important role in stimulating cellular metabolism in normal and cancer cells (35). The main source of energy in most cells is glucose, which enters the cell through specific transporters, particularly glucose transporter 1 (GLUT1) in glial cells (36). We studied GLUT1 mRNA expression after 24 h of IGF1 stimulation in U87Empty, U87WT, and U87Mut cells. Although the GLUT1 expression significantly increased in all cell lines (**Figure 3A**), the increment observed in U87WT cells was higher than that observed in U87Empty or IGF1R mutant-expressing cells (**Figure 3B**). In all cases, the increase was abrogated by preincubation with OSI906 for 60 min (**Figure 3A**).

Glioma cells can metabolize glucose into pyruvate and produce lactate through LDH (37). We analyzed the LDH activity and found that mutant cells had a lower activity in basal conditions compared to U87Empty and U87WT cells (**Figure 3C**). After 24 h of IGF1 stimulation, the LDH activity did not change in U87Empty cells but had a significant increase in WT and mutant clones that were abrogated with OSI906 (data not shown).

Pyruvate generated from glucose catabolism can also enter the mitochondria and be converted to acetyl-CoA. The key enzyme regulating this step is pyruvate dehydrogenase complex (PDC). The activity of this complex is regulated by phosphorylation mediated by pyruvate dehydrogenase kinases (PDKs) and phosphatases (PDPs), the active form of this complex being its non-phosphorylated form (38). We performed Western blot studies and found that the basal phosphorylation of the PDC in U87WT clones was lower than in U87Empty or in mutant IGF1R-expressing cells (**Figure 3D**). In all cases, stimulation with IGF1 decreased the PDC phosphorylation levels, suggesting an increment in its activity. The specificity of the response to IGF1 was confirmed by the use of OSI906, which restored the PDC phosphorylation status to basal conditions (**Figure 3D**).

Glucose oxidative metabolism is important in diverse cancers, including glioblastomas, since it allows TCA intermediates to be derived from different anabolic pathways (39). Additionally, fatty acids can act as critical bio-energetic substrates within glioma cells (11). The enzyme fatty acid synthase (FAS) catalyzes the synthesis of palmitic acid from malonyl- and acetyl-CoA substrates (40). Additionally, the enzyme long-chain acyl-CoA synthetase 5 (ACSL5) catalyzes the conversion of long-chain fatty acids to their active form acyl-CoAs for both the synthesis of cellular lipids and degradation *via* beta-oxidation (41). We studied FAS and ACSL5 expression by Western blot and found that, in U87WT clones, a small increment in FAS expression was observed under IGF1 stimulation in comparison with basal conditions, while this was not observed in U87Empty cell line or mutant clones (**Figure 3D**). As for ACSL5 expression, it was lower in U87Mut compared to U87WT or to U87Empty cells, and no changes were observed in response to IGF1 (**Figure 3D**).

Additionally, we decided to study mTOR activation, as this kinase is part of a complex responsible for the integration of multiple signals to regulate a wide variety of cellular functions, such as translation, transcription, protein turnover, and cell growth (42). To evaluate the mammalian target of rapamycin complex (mTORc) activation, we studied the S6K phosphorylation status in basal conditions or after 24 h of IGF1 stimulation (**Figure 3D**). We found that, in both conditions, S6K was phosphorylated in U87Empty cells and also had a higher intensity in U87WT clones. We were not able to detect S6K phosphorylation in mutant clones in any of the conditions studied.

**Figure 4:**
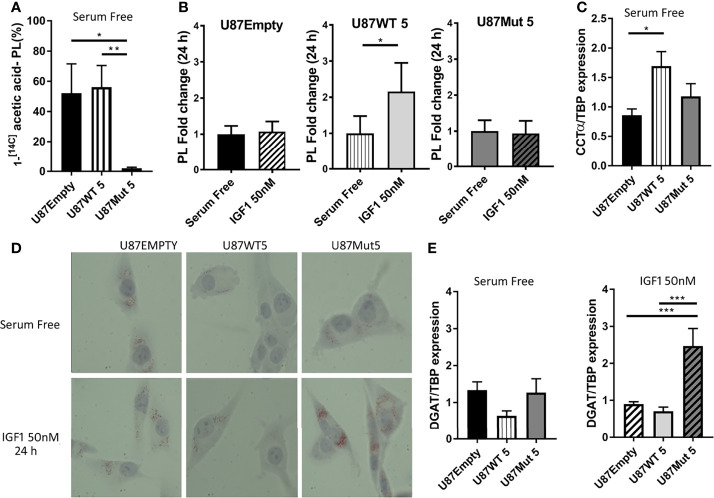
Effects of IGF1R localization over phospholipid and triglyceride synthesis. U87Empty, U87WT, and U87Mut cells were cultured 24 h in serum-free medium or with 50-nM IGF1 stimulation (IGF1) or pre-incubated (1 h) with 0.5 μM OSI906 (IGF1+OSI) in the presence of 1-^[14C]^ acetic acid. Lipids were extracted and resolved by thin-layer chromatography, and the radioactivity associated with each condition was quantified. Three independent experiments were performed. **(A)** 1-^[14C]^ acetic acid incorporated to newly synthesized phospholipids. The results are expressed as percentage of 1-^[14C]^ acetic acid incorporated into phospholipid fraction normalized by the number of cells seeded/well. Values are presented as mean ± SD (*p < 0.05, **p < 0.005, ANOVA, Tukey’s post-test). **(B)** Changes in the amount of phospholipids in response to 24-h IGF1 stimulation in each cell line. The results are presented as fold change compared to control conditions (serum-free). Values are expressed as mean ± SD (*p < 0.05, Mann–Whitney test). **(C)** U87Empty, U87WT, and U87Mut cells were cultured 24 h in serum-free medium. CCTα mRNA expression was calculated by rqPCR by the relative quantitation method, and the results are presented as fold change compared to U87Empty cells. Values are expressed as mean ± SD of three independent experiments performed in triplicates (*p < 0.05, ANOVA, Tukey’s post-test). **(D)** Representative picture of lipid droplets visualized by oil red O (ORO) in U87Empty, U87WT, and U87Mut cells after 24 h of incubation in serum-free medium or with 50-nM IGF1 stimulation (IGF1). **(E)** DGAT mRNA expression of U87Empty, U87WT 5, and U87Mut 5 cells incubated in serum-free medium (left) or with 50-nM IGF1 stimulation (IGF1) (right). The results are presented as fold change compared to U87Empty cells. Values are expressed as ± SD of three independent experiments performed in triplicates (***p < 0.001, ANOVA, Tukey’s post-test).

As we detected an increase in the expression or activation of several enzymes related to fatty acid biosynthesis in U87WT clones, we decided to deepen the study on this matter. Once fatty acids are synthesized, they can be converted into phospholipids which are incorporated to the cell membranes (43). We studied *de novo* phospholipid (PL) synthesis by 1-^[14C]^ acetic acid incorporation into lipid classes from U87Empty cells and U87WT and U87Mut clones in culture, using thin-layer chromatography. We found that the incorporation of 1-^[14C]^ acetic acid in the mutant clone was the lowest (**Figure 4A**). Additionally, after 24 h of IGF1 stimuli, PL synthesis was only increased in U87WT cells in comparison with basal conditions (**Figure 4B**). Next, we studied the expression of CTP-phosphocholine cytidylyl transferase-α (CCTα), a key enzyme involved in the synthesis of phosphatidyl choline. The results showed that, in basal conditions (FBS free medium), CCTα expression was significantly increased in the U87WT clone compared to the U87Empty cells (**Figure 4C**). IGFI stimulation did not modify the expression of the enzyme in any of the cells (data not shown).

On the other hand, in cases of lower metabolic activity, fatty acids can be esterified into triglycerides (TAGs) and stored as lipid droplets in the cytoplasm (44). We visualized the presence of lipid droplets using Oil Red O staining and found higher lipid storage in the cytoplasm of mutant IGF1R-expressing clones compared to U87WT and U87Empty cells (**Figure 4D**). Moreover, the storage was highly increased after 24 h of IGF1 stimulation in U87Mut clone (**Figure 4D**). We also studied DGAT1, an enzyme involved in TAG biosynthesis, and found that its mRNA expression was higher in U87Mut compared to U87WT cells, a difference that became statistically significant after 24 h of IGF1 stimulation (**Figure 4E**).

### Gene Expression Analysis Performed by Microarray Studies Supports Our *In Vitro* Experimental Results

Differential gene expression was assessed by microarray analysis. We compared U87WT cells *vs*. U87Mut RNA expression in basal conditions (FBS-free medium) and after 24 h of IGF1 stimulation (50 nM), with or without 1 h of preincubation with 0.5 μM OSI906. A list of 6,187 genes differentially expressed was obtained (**Supplementary Table S1**). The normalized expression data was further simplified to a trinary matrix with values of 1 (for overexpressed genes), 0 (no change), and -1 (underexpressed genes) using the fold change values. We obtained gene lists that are differentially expressed between experimental conditions (see “Materials and Methods”, section 1) and found 737 genes upregulated by IGF1, 1,945 genes downregulated by IGF1, and 182 genes overexpressed in all conditions. Next, we turned our attention to those genes related to our *in vitro* findings, focusing on metabolism. The results are presented in **Table 1**. Additionally, we used these lists as input for the DAVID database (DAVID 6.8 Oct. 2016 version) to identify enriched clusters of genes belonging to functionally related groups which are determinants of the observed *in vitro* phenotypic traits. We found several enriched clusters, 2 of which involved differentially expressed genes under IGF1 stimulation: 19 belonging to the oxidative phosphorylation process and 31 grouped under the metabolic pathways category. Another cluster involved genes belonging to lipid metabolism.

**Table 1:** Genes related to metabolism over- or underexpressed in U87WT *vs*. U87Mut cells.

Gene	OMIM	Gen name
Overexpressed in U87WT clone after 24 h of IGF1 stimulation		
*GAPDH*	* 138400	Glyceraldehyde-3-phosphate dehydrogenase
*SLC2A11*	* 610367	Solute carrier family 2 (facilitated glucose transporter), member 11
*LDHB*	* 150100	Lactate dehydrogenase B
*LPIN1*	* 605518	Lipin 1
*MDH1*	* 154200	Malate dehydrogenase 1, NAD
*LDHA*	* 150000	Lactate dehydrogenase A
*PCK2*	* 614095	Phosphoenolpyruvate carboxykinase 2
*IDH3A*	* 601149	Isocitrate dehydrogenase 3 (NAD+) alpha
*PPAP2C*	* 607126	Phosphatidic acid phosphatase type 2C
Underexpressed in U87WT clone after 24 h of IGF1 stimulation		
*ACAT1*	* 607809	Acetyl-CoA acetyltransferase 1
*ACSF3*	* 614245	Acyl-CoA synthetase family member 3
*CPT1A*	* 600528	Carnitine palmitoyltransferase 1A
*PDPR*	* 617835	Pyruvate dehydrogenase phosphatase regulatory subunit
*SLC2A14*	* 611039	Solute carrier family 2 (facilitated glucose transporter), member 14
*FABP5*	* 605168	Fatty acid-binding protein 5
*AGPAT1*	* 603099	1-Acylglycerol-3-phosphate O-acyltransferase 1
*SLC16A3*	* 603877	Solute carrier family 16 (monocarboxylate transporter), member 3
*PFKFB4*	* 605320	6-Phosphofructo-2-kinase/fructose-2,6-biphosphatase 4
*FADS3*	* 606150	Fatty acid desaturase 3
*LDHAL6A*	* 618928	Lactate dehydrogenase A-like 6A
*ACSL6*	* 604443	Acyl-CoA synthetase medium-chain family member 6
*ACSF2*	* 610465	Acyl-CoA synthetase family member 2
*ACSL5*	* 605677	Acyl-CoA synthetase long-chain family member 5
*PFKFB2*	* 171835	6-Phosphofructo-2-kinase/fructose-2,6-biphosphatase 2
*GCK*	* 138079	Glucokinase
*SPTLC3*	* 611120	Serine palmitoyltransferase, long-chain-base subunit 3
Overexpressed in U87WT clone in all conditions		
*CH25H*	* 604551	Cholesterol 25-hydroxylase
*LPCAT3*	* 611950	Lysophosphatidylcholine acyltransferase 3
*SREBF1*	* 184756	Sterol regulatory element-binding transcription factor 1
*SOD2*	* 147460	Superoxide dismutase 2, mitochondrial
*SOD1*	* 147450	Superoxide dismutase 1, soluble

The numbers displayed in table 1 are OMIM numbers. An asterisk (*) before an entry number indicates a gene.

### IGF1R Nuclear Localization in Glioma Cells Shortens Tumor Latency and Contributes to Tumor Growth

**Figure 5:**
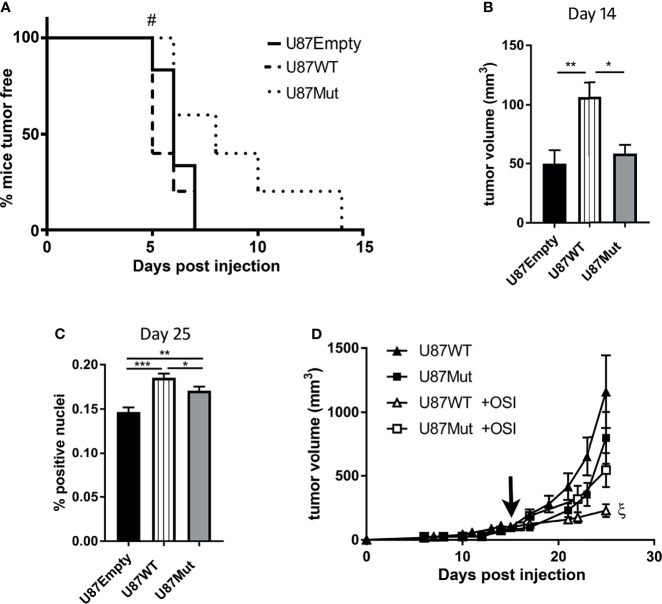
Effects of IGF1R localization in glioblastoma xenografts. **(A)** Incidence and latency period of tumors developed by U87Empty (*n* = 5), U87WT (*n* = 5), and U87Mut (*n* = 5) cells were recorded and plotted in a Kaplan–Meier curve (#*p* < 0.05, log-rank test). **(B)** Tumor growth: tumor volume was calculated for each developed tumor. The two longest perpendicular axes in the x/y planes were measured, and the volume was calculated 14 days after U87Empty, U87WT, or U87Mut cell injection. Values are presented as mean ± SD (*p < 0.05, **p < 0.005, ANOVA, Tukey’s post-test). **(C)** Proliferation index *in vivo*. The mice were injected with BrdU 3 h before sacrifice. The tumor-positive nuclei were quantified. The results are expressed as mean ± SD (*p < 0.05, **p < 0.005, ***p < 0.001, ANOVA, Tukey’s post-test). **(D)** The mice injected subcutaneously with U87WT or U87Mut clones were treated orally with OSI906 when the tumor volume reached 150 mm^3^ (black arrow). The results are expressed as mean ± SD [ξ*p* < 0.05 U87WT (closed triangles) *vs*. U87WT +OSI (open triangles)].

To study the effect of IGF1R subcellular localization *in vivo*, using subcutaneous xenografts as a proof of concept, 1.5 × 10^6^ U87Empty, U87WT, or U87Mut cells were injected subcutaneously in immunodeficient male mice [N:NIH (S)- Fox 1^nu^]. As shown in **Figure 5A**, the period until 50% of the tumors in each group could be measured using a caliper was 1 day shorter when the mice were injected with U87WT clones compared to the ones injected with U87Empty cells (5 *vs*. 6 days). On the contrary, this period was 2 days delayed for tumors developed by U87Mut clones (*p* < 0.05, log-rank test). None of the mice injected remained tumor-free at 14 days after injection (**Figure 5A**).

Additionally, tumors developed by U87WT clones grew faster (**Figure 5B**) and reached bigger volumes, as early as day 14, compared to the ones developed by U87Empty or U87Mut cells (**Figure 5C**). The tumor cell proliferation rate, depicted as *in vivo* BrdU incorporation, was also higher in U87WT tumors compared to the ones established by U87Empty cells or mutant clones at the end of the study (**Figure 5C**).

Finally, the mice were orally treated with OSI906 when tumors developed from U87WT or U87Mut clones reached a volume of 150 mm^3^ approximately (day 16 or 17 post-injection for U87WT or U87Mut, respectively). We found that the inhibitor was effective in delaying tumor growth solely on U87WT xenografts (**Figure 5D**).

## Discussion

The present study was designed to explore the effects of IGF1R nuclear localization previously observed in pediatric high-grade gliomas. To this end, we used the human glioblastoma U-87 MG (U87) cell line to generate IGF1R-overexpressing cells with or without the receptor’s capacity to translocate to the nucleus.

The cell line chosen for our experiments was derived from an adult grade IV glioma, and even though its usefulness as a glioma model has been questioned, an agreement has been reached about its glioma-like characteristics (45). The adult origin of this cell line is one of the limitations of our study. However, it has been reported that IGF-IR is overexpressed in the majority of adult GBMs compared with the normal brain, though the nuclear localization of IGF1R was not analyzed. With regard to standard clinical factors, this overexpression was associated with an independent prognostic value in terms of cancer-specific survival, suggesting that IGF-IR could be an interesting target for GBM therapy in adult patients (46). Additionally, U87 cells present wild-type isocitrate dehydrogenase (IDH) enzyme, which is a characteristic of pediatric-type, diffuse, low- and high-grade gliomas (6), while in adult gliomas, adult-type diffuse gliomas, mutations in IDH are observed (6). Moreover, this cell line has an inactivating mutation in PTEN, such that it has also been described for pediatric glial tumors (47, 48).

To model a cell line in which IGF1R fulfills its actions as a transmembrane receptor but is not able to translocate to the nucleus, we performed directed mutagenesis against 3 specific lysins (Lys1025, Lys1100, and Lys1120) in a vector containing a fully mature IGF1R protein coding sequence. When these 3 lysin residues are replaced by arginine residues, the receptor cannot be SUMOylated; hence its nuclear translocation is prevented (22). It is important to highlight that, although those 3 amino acid residues are in the receptor’s tyrosine kinase domain, its tridimensional structure is not seriously affected, and the IGF1R autophosphorylation capacity remains intact (22).

In a previous study, we analyzed the mRNA expression of IGF1R in pediatric low- and high-grade gliomas (27). Considering these data, we selected U87WT and U87Mut clones as the ones which better represented high-grade gliomas, regarding the mRNA expression of IGF1R. No differences were also observed in the expression of IGF1, IGF2, and IR, supporting the concept that changes in cell phenotype could only be attributed to IGF1R capability to translocate to the nucleus.

As expected, in both U87WT and U87Mut clones, IGF1R phosphorylation and its two main downstream pathway activation (PI3K/AKT and MAPK) were conserved. Unlike AKT, ERK phosphorylation seems to be basally activated and increased when stimulated with IGF1. This phenomenon has already been described in U87 cell line and is attributed to PTEN enzyme mutation. The authors described that, when wild-type PTEN expression is restored in these cells, IGF1 sensitivity is reestablished (49). Additionally, after 8 h of IGF1 stimulation (22), we were able to verify IGF1R nuclear translocation only in U87WT clones.

Dual IGF1R/IR inhibitor OSI906 was used to study the specificity of the response to IGF1 in both receptor signaling and translocation experiments. The decrease in IGF1R activation and nuclear localization when cells were pre-incubated with OSI906 revealed that receptor phosphorylation is a necessary condition for its translocation to the nucleus as has been described by Aleksic and colleagues (21). These results altogether support the use of these cell lines as suitable models to carry on our experimental work.

Insulin-like growth factors play important roles in stimulating cell proliferation (13). It is also known that one of IGF1R’s effects in the nucleus is its association with LEF1 transcription factor, which leads to an increased expression of specific genes, like Cyclin D1, involved in cell cycle progression (50). Accordingly, in a previous study, we have described an increment in Cyclin D1 expression in pediatric gliomas where IGF1R had nuclear compared to non-nuclear localization (27). In the present work, the addition of IGF1 failed to stimulate the cellular proliferation of U87Empty, U87WT, and U87Mut clones in culture. No changes in cell cycle phase distribution were observed either, regardless of the capacity of IGF1R to translocate to the nucleus. It is important to consider that, in these *in vitro* experiments, IGF1 is the only growth factor added to the serum-free culture medium. Other growth factors, such as nerve growth factor (NGF), are essential for cell growth and differentiation in the nervous system. NGF has recently been reported to stimulate U87 cell line proliferation in a dose-dependent manner (51). It also has synergistic effects with IGF1 in the SNC (52). In this scenario, we cannot rule out the possibility that the presence of other growth factors besides IGF1 may be needed to stimulate U87 cell proliferation in culture.

Concerning cell migration, even though gliomas do not metastasize outside of the SNC (3), during their growth, cells may migrate along the optic tract to the hypothalamus and brain stem where total resection is very difficult (53). The IGF system has known effects in promoting cancer cell migration (34). Besides that, IGF1R has been related to the epithelial–mesenchymal transition process (EMT), contributing to metastasis formation and drug resistance (54). In our study, after 36 h of IGF1 stimulation, we observed an increase in cell migration only in U87WT5 clones, compared to control conditions. This result may be indicating that not only IGF1R overexpression is a requirement to induce migration but also its capacity to migrate to the nucleus is needed to achieve specific effects. Regarding the receptor nuclear localization, one of the genes reported to be overexpressed after IGF1R interaction with LEF1 in the nucleus is Axin2 (50), an expression which we also found to be increased in U87WT clones in basal conditions (**Supplementary Table S1**). Axin2 is a scaffold protein that is a direct target of the Wnt/β-catenin pathway. Its overexpression has already been related to increased migration in Wilms tumor (55) and the promotion of the EMT program in colon carcinoma cells (56). Therefore, it is possible that, in our model, this gene is also responsible, at least in part, for the observed phenotype.

Our study also focused on cell metabolism, as IGFs are involved in metabolic regulation, a key factor for cancer cells to sustain growth and proliferation (35). It is known that IGF1 has direct effects on stimulating glycolytic flux favoring tumor cell energy production, acting through IGF1R to increase the GLUT1 expression (57), the main glucose transporter in glial tumors (36). In our study, we found an increase in GLUT1 expression after 24 h of IGF1 stimulation in all cell lines, this increase being higher in U87WT clones. We hypothesized that there is a dual effect mediated by IGF1 stimulus: on the one hand, increased GLUT1 expression in all cell lines is mediated by IGF1R canonical actions as a membrane tyrosine kinase receptor, considering previous evidence that GLUT1 expression may be increased after the activation of the PI3K/AKT pathway (58); on the other hand, in U87WT clones, an additive effect may be taking place due to IGF1R nuclear localization. Recent evidence shows that, in the nucleus, IGF1R can interact with specific regulatory regions and, by RNA polymerase II recruitment, upregulate the expression of specific genes, like JUN and FAM21A (59). JUN is a pro-oncogenic gene which mediates cellular processes like proliferation and survival, and its upregulation in response to IGF1 stimulation has already been described (60). As a transcription factor, JUN increases the expression of transforming growth factor-β (TGF-β) (61). Although our microarray result analysis did not show an increased JUN expression, a higher TGF β1 expression was detected for U87WT clone in all conditions studied (**Supplementary Table S1**). In gliomas, TGFβ1 stimulation has been related to increased glucose uptake, glycolytic flux, and lactate production mediated, at least in part, by an increment in GLUT1 expression (62). On the other hand, FAM21A functions as an integral component of the Wiskott–Aldrich syndrome protein and SCAR homolog (WASH) that has been reported as a regulator of NF-κB gene transcription (63). Interestingly, it has been described that, in brain tumors, NF-κB signaling also promotes glucose uptake by upregulating the GLUT1 expression (64). In our gene expression studies, not FAM21A but FAM21C, another subunit of the WASH complex, was found to be increased in U87WT cells in basal conditions (**Supplementary Table S1**). Therefore, it is possible that similar regulatory mechanisms may occur in our model.

Even in the presence of oxygen, many cancer cells, including gliomas, have long been thought to metabolize glucose to pyruvate and then generate lactate *via* LDH enzyme for energy production (37). This aerobic glycolysis process, known as the “Warburg effect”, is a characteristic of cancer cells, and even though it only renders low ATP production, it favors TCA cycle intermediates to be diverted to macromolecular precursor synthesis (39). It is known that LDH enzyme expression is regulated by multiple factors both transcriptionally and translationally (37). In our work, we studied LDH activity in all cells and found that, in basal conditions, the lowest activity was registered for U87Mut clones. This result suggests that IGF1R nuclear localization is needed to maintain LDH activity at similar levels as the parental cells. At this point, we do not rule out the possibility of an alternate mechanism involving transcriptional regulation for the expression of LDH enzyme mediated directly or indirectly by nuclear IGF1R. On the other hand, when cells were stimulated with IGF1 for 24 h, an increase in LDH activity was observed only in IGF1R-overexpressing clones (U87WT and U87Mut), but not in U87Empty cells. The microarray analysis showed that both LDHA and LDHB genes (that encode all LDH subunits) were found overexpressed in U87WT clone after 24 h of IGF1 stimulation (**Table 1**). Hence, there seems to be another effect related to IGF1R actions as a transmembrane receptor enhanced by its overexpression but not related to its nuclear localization. In fact, similar effects were mediated after the activation of fibroblast growth factor receptor 1, which is also a tyrosine kinase receptor (65).

Simultaneously, glucose incorporated to the cell may also be diverted to the process of oxidative phosphorylation, where pyruvate is able to enter the TCA cycle converted into acetyl-CoA. This critical step is mediated by the pyruvate dehydrogenase complex (PDC), whose activation in its unphosphorylated form is tightly regulated (38). In our study, we found that, in basal conditions, the U87WT clone was the one which registered the lowest phosphorylation. Although after 24 h of IGF1 stimulus, an increase in PDC activity (depicted as a decline in its phosphorylated form) was observed for all cell lines, the differences were still evident. A dual process seems to be taking place again: on the one hand, the activation of PDC after IGF1 stimulation is independent of IGF1R cellular localization. On the other hand, there seems to be a role for nuclear IGF1R in the activation of the enzyme in basal conditions that could provide an explanation for the initial lower phosphorylation observed in U87WT clone. This process may be involving changes in PDC-positive (PDPs) and PDC-negative (PDKs) regulators that need to be studied in detail. The preliminary results from the microarray data analysis showed a decrease in PDK1 expression in U87WT clone in both basal and stimulated conditions (**Supplementary Table S1**). Moreover, we also detected an under-expression of the PDPR gene in U87WT clone after 24 h of IGF1 stimulation (**Table 1**). This enzyme decreases the sensitivity of the PDP catalytic subunit; hence, its lower expression may imply a higher PDP activity and a lower PDC phosphorylation rate.

As we have mentioned before, when metabolic reprogramming occurs in cancer cells, TCA cycle intermediates may be diverted to different anabolic pathways, including fatty acid biosynthesis (39). In fact, enhanced lipid biosynthesis is closely linked to tumorigenesis, and as fatty acid supply is highly dependent on *de novo* synthesis, several enzymes in the lipid biosynthesis pathways are considered to be directly involved in tumor cell survival (40).

In our model, we studied fatty acid synthesis focusing on the expression of 2 specific enzymes: FAS, responsible for catalyzing the last step of palmitic acid synthesis (40), that is expressed in gliomas, and its expression is known to correlate with tumor grade (66), and ACSL5, in charge of fatty acid activation (41). A lower expression of both enzymes was observed for U87Mut clones in comparison with U87Empty and U87WT cells. Moreover, the microarray study showed a higher expression not only of ACSL5 but also of ACSF2 and ACSM6 in the U87WT clone in basal conditions (**Supplementary Table S1**), a difference that is lost after 24 h of IGF1 stimulation (**Table 1**). These results suggested that fatty acid synthesis may be higher when IGF1R is able to translocate to the nucleus. In hepatocellular carcinoma cells, high levels of TGF-β have been related to a higher expression of enzymes involved in free fatty acid synthesis such as ACSL5 and significantly higher amounts of phospholipid levels (67). Furthermore, IGF1 has already been described as an inductor of the expression of several genes related to fatty acid synthesis (68). This process is mediated mainly through sterol response element-binding protein-1 (SREBP-1/SREBF1) acting as a transcription factor (69). Particularly, FAS and ACSL5 expression is known to be upregulated transcriptionally, at least in part, because of the existence of SREBF1 binding sites on their gene’s promoter (70, 71). In concordance with this, our gene expression analysis found SREBF1 to be overexpressed in U87WT clone in all the conditions studied (**Table 1**). Even so, the mechanism by which nuclear IGF1R contributes to increase fatty acid synthesis in our model still needs to be studied properly to be understood.

Once synthesized, fatty acids may play diverse and important roles in the function of cancer cells. Regarding this point, recent studies have demonstrated that glioma cells primarily use fatty acids as a substrate for energy production (11). It is known that bio-energetic pathways in glioma cells do not occur in isolation. In fact, there is a mechanistic link between beta-oxidation and aerobic glycolysis, which explains that nonoxidative glycolysis can occur alongside the oxidation of substrates, like fatty acids, providing significant amounts of acetyl-CoA for ATP production (10).

Additionally, fatty acids play critical anabolic roles within the cell, as they can be transformed into phospholipids to be incorporated in the plasma membrane (43). In our work, we have found that, in basal conditions, the highest expression of CCTα enzyme, which is involved in phosphatidyl choline synthesis, was detected for U87WT clones. Although CCTα expression is enhanced by diverse transcription factors such as E2F (72) that is regulated by IGF1 stimulation (60), we do not discard other possible mechanisms triggered by IGF1R nuclear localization to justify the higher expression of CCTα detected in U87WT cells. Additionally, this higher expression may be responsible for the increased phospholipid synthesis observed after 24 h of IGF1 stimulation only in this clone, but not in U87Empty or U87Mut cells.

When cells present a lower metabolic activity, fatty acids can be shuttled into lipid droplets as TAGs and be stored in the cytoplasm as energy reservoir (44). We found that, even though the IGF1 stimulus increased the lipid droplet accumulation in all cells, this increase was higher in U87Mut clones, those which also registered a higher lipid storage in basal conditions. Consistent with this result, the expression of DGAT1, involved in TAGs biosynthesis, was also higher in U87Mut clones after IGF1 stimulation. Lipid droplet biogenesis and metabolism have been linked to carcinogenesis, both as a reservoir of biosynthetic substrates and as regulators of lipid signaling molecule availability (73). Our results are consistent with the proposed functions of lipid droplets in cancer because the U87Mut clone showed a higher lipid droplet content and a lower metabolic activity.

To have a glance on the overall metabolic state of U87Empty and IGF1R-overexpressing clones, we decided to study the mTOR complex functionality. mTORc integrates signaling from growth factor pathways with cellular energy and nutrient levels to coordinate the cell biosynthetic machinery (42). Particularly, protein translation is started after S6K and 4E-BP1 phosphorylation mediated by mTORc1, a process stimulated by different growth factors, including IGF1 (74). In our model, in both basal conditions and after IGF1 stimulation, we observed a higher S6K phosphorylation status in U87WT clones compared to U87Empty cells, suggesting increased mTORc1 activation. Interestingly, we were not able to detect S6K in its phosphorylated form in U87Mut clone, which would suggest a lower activity of mTORc1 in this cell line and hence a lower protein translation rate.

Taken together, our results show that, in U87WT clones, fatty acid and phospholipid synthesis were higher in comparison to U87Mut clones, which presented a higher lipid droplet accumulation rate instead. Thus, the nuclear localization of IGF1R would be playing a role in increasing anabolic pathways and maintaining cell growth, processes that require a sustained energy input possibly supported by a higher beta-oxidation rate. Accordingly, when the results from the microarray were analyzed using DAVID database, the oxidative phosphorylation process cluster was found to be enriched in U87WT clone. We hypothesized that a higher oxidative metabolism in this context may be due to an increase in the beta-oxidation rate produced when IGF1R is able to translocate to the cell nucleus. On the other hand, even if to a lesser extent, mutant clones present active fatty acid biosynthesis, suggesting that the inability of IGF1R to translocate to the nucleus would lead to a decreased metabolic activity of U87Mut cells, which may provoke an accumulation of synthetized lipids in the cytoplasm.

Knowledge about the actions carried out by nuclear IGF1R is scarce. Recent reports have demonstrated that, once in the nucleus, IGF1R can interact with specific regulatory regions and upregulate the expression of specific genes related to diverse cellular processes (21, 22, 50, 59). Hence, it is possible that the presence of IGF1R in the nucleus of U87WT cells led to the regulation of genes that would allow us to explain the differences in phenotype observed in U87WT compared to U87Mut cells. On the other hand, receptor internalization has been regarded as a mechanism required for receptor degradation, usually in endosomes and lysosomes. However, at the present time, it is clear that receptor endocytosis is crucial for the formation of signalosomes and for the regulation of hormonal signals (75). Indeed internalized receptors are capable of linking different lysosomal and recycling paths, resulting in the enhancement or attenuation of the hormonal signal (76). A recent study (77) demonstrated that phosphorylation of specific IGF1R residues (Tyr1250/1251) is required for the translocation of IGF1R to the Golgi apparatus, where it might contribute to invasiveness. We did not test the status of Tyr1250/1251or any other specific IGF1R residue in our model. As of today, there is no evidence that IGF1R translocation to or accumulation in the Golgi apparatus regulates lipid metabolism or inhibits cell proliferation/survival signals of glioma cells. However, we cannot rule out that a possible accumulation of IGF1R in the cytoplasm of U87Mut cells is having a potential role in the observed phenotype. The novelty and complexity of the interplay between the classical regulation of genes resulting from the signaling that follows the activation of membrane-based IGF1R, internalization, trafficking, and the recently described gene regulation provoked by the presence of IGF1R in the nucleus of the cells merit further investigation.

When injected subcutaneously in immunodeficient mice, all U87Empty cells and IGF1R-overexpressing clones were able to develop tumors. Interestingly, those generated from U87WT cells had a shorter latency period and reached larger volumes than the ones that arise from the injection of U87Empty and U87Mut cells. In this scenario, we discarded the possibility that the time of appearance and the size of these tumors relied only on IGF1R overexpression, the receptor nuclear translocation being a key factor in this matter. Although these results are not consistent with *in vitro* proliferation studies, it is necessary to consider that cultured cells were only stimulated by IGF. Therefore, we interpreted that the availability of growth factors/mitogens that are present *in vivo* may be responsible for the differences in growth of the observed tumors.

To study the effects of a possible treatment for glioblastomas involving the inhibition of IGF1R signaling, we repeated the injection of immunodeficient mice with U87WT and U87Mut clones, and when a tumor developed, we treated the mice with OSI906. This drug, commercially known as linsitinib, is an orally bioavailable, selective, dual IR/IGF1R inhibitor which has been tested clinically in different cancers (28). The present study shows that treating mice with OSI906 was only effective for tumors generated by U87WT clones, where IGF1R is able to translocate to the nucleus. Since OSI906 is a dual inhibitor, it is important to emphasize that U87WT and U87Mut clones have a comparable IR expression, and that is 3 times lower than IGF1R expression. Thus, we assumed that the different effects observed between clones after treatment with OSI906 resulted from IGF1R rather than IR inhibition.

Previous studies conducted by Seely and colleagues (78) showed that, when U87 cells were injected subcutaneously in immunodeficient mice, the inhibition of IGF1R using a blocking antibody reduced the number of tumors to 60%. In the cases that tumors managed to develop, treatment reduced their growth and increased intratumor apoptosis. These results highlighted the importance of IGF1R signaling in glioma development and growth but made no distinction on receptor intracellular localization. We are aware of the limitation of the *in vivo* model because a heterotypic location was used for our studies. However, in this work, we have proved that inhibition of IGF1R using OSI906 prevents not only its actions as a tyrosine kinase receptor but also its nuclear relocation. Considering that IGF1R’s presence in the cell nucleus causes subcutaneous tumors to develop earlier and to reach larger volumes in addition to making them sensitive to the target therapy, it is possible that the receptor plays essential roles in the nucleus, directly or indirectly stimulating the expression of genes related to the observed phenotype.

## Conclusions

As a summary, we presented evidence that IGF1R nuclear localization stimulates cell motility and metabolism without affecting the cell proliferation of cultured glioma cells. *In vitro*, the increase in metabolic activity depicted as a higher expression of the glucose transporter and stimulation in lipid biosynthesis and usage—in detriment of its accumulation—would also be favored in this scenario. *In vivo*, the metabolic advantage given by IGF1R’s capacity to translocate to the nucleus not only allows a higher proliferation rate and the earlier development of tumors but also renders the cells sensitive to a targeted therapy with OSI906. The complex interactions between the canonical IGF1R and the emerging IGF1R signaling pathways that regulate diverse cellular functions, along with clinical and therapeutic implications, need to be critically assessed.

## Data Availability Statement

The datasets presented in this study can be found in online repositories. The names of the repository/repositories and accession number(s) can be found below: https://www.ncbi.nlm.nih.gov/geo/query/acc.cgi?acc=GSE195896.

## Ethics Statement

The animal study was reviewed and approved by CICUAL—Comité de Docencia del Hospital de Niños Dr. R. Gutiérrez.

## Author Contributions

AM, FC, and PP contributed to conceptualization. AM, MCF, EC, and MG contributed to the methodology. AM, MCF, and EC contributed to validation. AM, MCF, MG-B, and PP contributed to formal analysis. FC, MV, and MGL contributed to investigation. IB and MGL contributed to the resources. CS, AB, and MM contributed to microarray data processing. AM and MCF contributed to data curation. AM, MCF, and EC contributed to writing—original draft preparation. PP, MCF, and MG-B contributed to writing—review and editing. AM, MCF, and PP contributed to visualization. PP contributed to supervision, project administration, and funding acquisition. All authors contributed to the article and approved the submitted version.

## Funding

This research was funded by Instituto Nacional del Cancer, Ministerio de Salud, Argentina (grant 2016–2018, awarded to PP). EMD Millipore Charitable Contribution Agreement for Healthcare Organizations 2022 was the funding source for publication fees.

## Conflict of Interest

The authors declare that the research was conducted in the absence of any commercial or financial relationships that could be construed as a potential conflict of interest.

## Publisher’s Note

All claims expressed in this article are solely those of the authors and do not necessarily represent those of their affiliated organizations, or those of the publisher, the editors and the reviewers. Any product that may be evaluated in this article, or claim that may be made by its manufacturer, is not guaranteed or endorsed by the publisher.
