# Supplementary figures and images for: Type 1 Insulin-Like Growth Factor Receptor Nuclear Localization in High-Grade Glioma Cells Enhances Motility, Metabolism, and In Vivo Tumorigenesis
Source: Front Endocrinol (Lausanne). 2022 Apr 27;13:849279. doi: 10.3389/fendo.2022.849279 (PMC9094447; doi:10.3389/fendo.2022.849279)

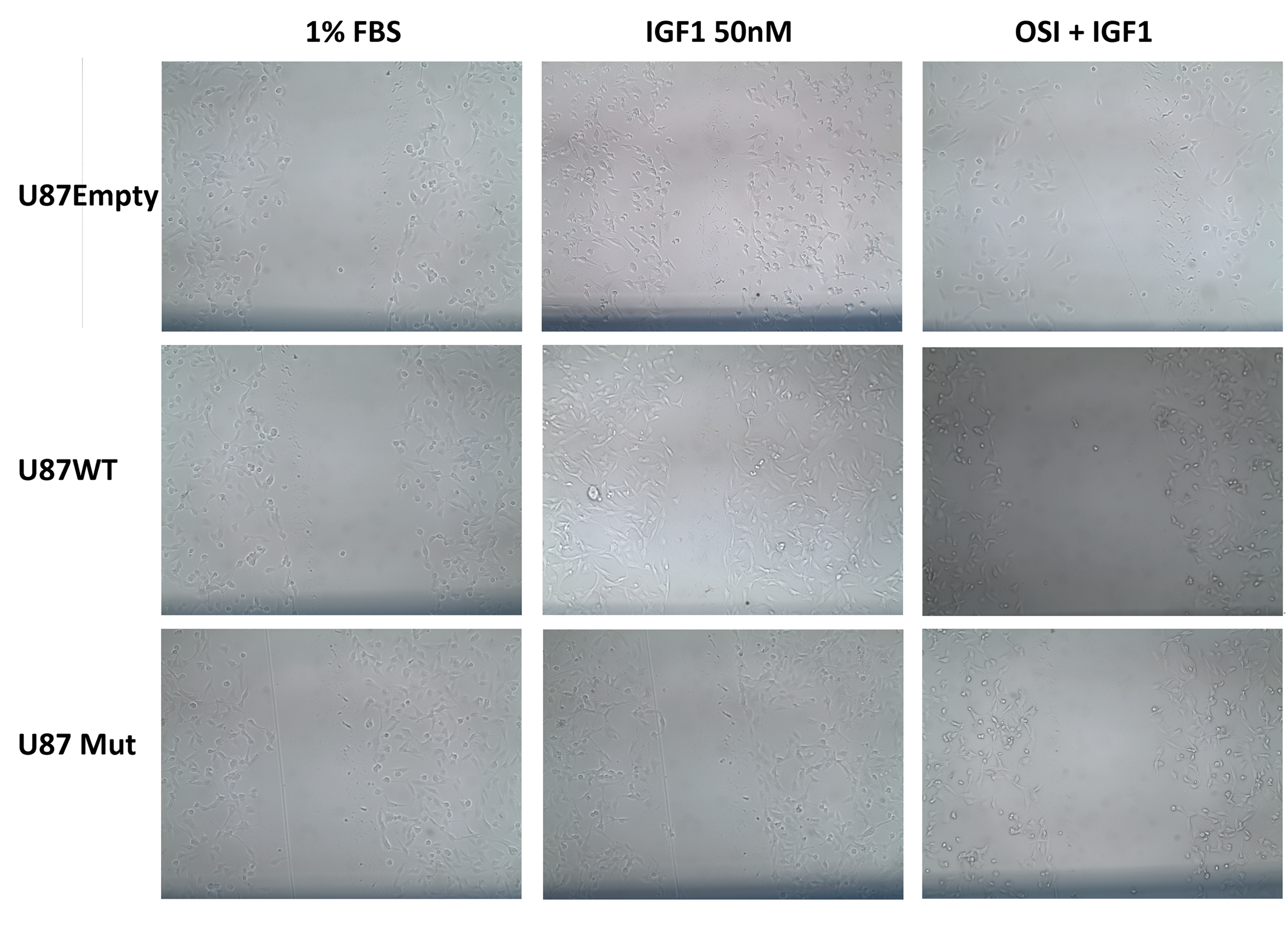

Supplement: Supplementary file 1 [file Image_1.tif]
